# Association of smoking habits with *TXNIP* DNA methylation levels in leukocytes among general Japanese population

**DOI:** 10.1371/journal.pone.0235486

**Published:** 2020-07-01

**Authors:** Keisuke Maeda, Hiroya Yamada, Eiji Munetsuna, Ryosuke Fujii, Mirai Yamazaki, Yoshitaka Ando, Genki Mizuno, Hiroaki Ishikawa, Koji Ohashi, Yoshiki Tsuboi, Shuji Hashimoto, Nobuyuki Hamajima, Koji Suzuki

**Affiliations:** 1 Department of Preventive Medical Sciences, Fujita Health University School of Medical Sciences, Toyoake, Japan; 2 Department of Hygiene, Fujita Health University School of Medicine, Toyoake, Japan; 3 Department of Biochemistry, Fujita Health University School of Medicine, Toyoake, Japan; 4 Department of Medical Technology, Kagawa Prefectural University of Health Sciences, Takamatsu, Japan; 5 Department of Biomedical and Analytical Sciences, Fujita Health University School of Medical Sciences, Toyoake, Japan; 6 Department of Healthcare Administration, Nagoya University Graduate School of Medicine, Nagoya, Japan; International University of Health and Welfare, School of Medicine, JAPAN

## Abstract

Thioredoxin-interacting protein (TXNIP) inhibits the activity of thioredoxin (TXN), leading to increased oxidative stress. Expression of the *TXNIP* gene is regulated by DNA methylation. However, no study has reported the influence of lifestyle factors on *TXNIP* DNA methylation. Our goal was to determine the association between smoking habits and *TXNIP* DNA methylation levels in a Japanese population. We conducted a cross-sectional study of 417 subjects (180 males and 237 females) participating in a health examination. We used a pyrosequencing assay to determine *TXNIP* DNA methylation levels in leukocytes. The mean *TXNIP* DNA methylation level in current smokers (75.3%) was significantly lower than that in never and ex-smokers (never: 78.1%, *p* < 0.001; ex: 76.9%, *p* = 0.013). Multivariable logistic regression analyses showed that the OR for *TXNIP* DNA hypomethylation was significantly higher in current smokers than that in never smokers, and significantly higher in current smokers with years of smoking ≥ 35 and Brinkman Index ≥ 600 compared to that in non-smokers. In conclusion, we found that current smokers had *TXNIP* DNA hypomethylation compared to never and ex-smokers. Moreover, long-term smoking and high smoking exposure also were associated with *TXNIP* DNA hypomethylation.

## Introduction

Thioredoxin (TXN) is a ubiquitous thiol-active protein that is expressed in many organisms. The protein contributes to cellular redox reactions that protect cells from oxidative stress, and TXN expression typically is induced following exposure to reactive oxygen species (ROS) [1, 2]. Thioredoxin-interacting protein (TXNIP) is a TXN-binding protein that inhibits the activity of TXN, thereby influencing balance of the cellular redox state [3]. TXNIP mRNA expression also is induced in response to glucose elevation and plays an important role in pancreatic β-cell function and glucose homeostasis [4]. TXNIP has been implicated in metabolic control including insulin release, glucose production, and glucose uptake from peripheral tissues [5, 6].

Recent studies have shown an association between DNA hypomethylation of the *TXNIP* gene and type 2 diabetes mellitus (T2DM) [7–10]. DNA methylation involves the transfer of a methyl group to carbon 5 of the cytosine base, yielding 5-methylcytosine. DNA methylation represses gene transcription by preventing the binding of transcription factors while also recruiting proteins with affinity for methylated DNA [11]. Indeed, epigenetic modifications such as DNA methylation have been shown to be associated with particular pathologies such as cancer, cardiovascular disease, and metabolic disease [12–14]. Previous research has established that changes in DNA methylation can occur in response to environmental factors [15] and lifestyles [16–18].

Smoking is an important lifestyle factor that alters the DNA methylation pattern. Changes in DNA methylation patterns due to smoking have been hypothesized to lead to changes in gene expression and to be involved in the development or progression of various diseases [19]. Some novel smoking-associated changes in DNA methylation have been identified by genome-wide methylation studies [20–22]. Most of the affected genes have been implicated in the development of smoking-related diseases [23].

Smoking increases oxidative stress and inflammation, leading to increased risks of health problems [24]. A recent study reported that cigarette smoke extract increased the levels of TXNIP in MIN6 pancreatic β-cells and the levels of TXNIP may be higher in the sera of current smokers compared to those in non-smokers [25]. Although it has been suggested that smoking affects DNA methylation at the *TXNIP* gene, there have been (to our knowledge) no epidemiological studies testing changes in DNA methylation of the *TXNIP* gene among smokers. Hence, we conducted a cross-sectional study to investigate whether smoking habits are associated with *TXNIP* DNA methylation levels in the general Japanese population.

## Methods

### Study participants

The present epidemiological study is part of the ongoing Yakumo Study, a population-based health examination conducted in Yakumo, a town located in the prefecture of Hokkaido, which lies in the northern part of Japan. A total of 525 subjects participated in a health examination at the end of August 2015. Among the participants, we excluded 27 individuals who did not provide written informed consent for the present study, 32 who did not complete the self-administered questionnaire, 20 who had samples that could not extract enough genomic DNA because of inadequate peripheral blood samples and 2 who had extremely high (>95%) or low (<3%) level of the *TXNIP* DNA methylation due to technical problems during the bisulfite conversion and pyrosequencing assay process. We also excluded another 27 individuals who had a clinical history of cancer. Thus, we analyzed a total of 417 subjects (180 males and 237 females). The protocol for this study was approved by the Ethics Committee of Fujita Health University (Approval No. 164).

### Collection of lifestyle information

Health information was obtained from the participants by trained public health nurses at the health examination. A self-administered questionnaire was used to collect lifestyle data such as smoking habits, alcohol consumption (current, ever, or never), and medical history of cancer (yes or no). Regarding smoking habit, participants were categorized into three categories as follows; current smokers: participants who currently smoke every day or sometimes, ex-smokers: participants who had smoked in the past but have quit, never smokers: participants who have never smoked in the past. The questions regarding smoking status also included age at which smoking started and the number of cigarettes smoked per day. The cumulative amount of cigarette consumption was evaluated by the Brinkman Index (BI) based on the self-administered questionnaire. The BI was determined as the number of cigarettes per day multiplied by years of smoking [26]. Anthropometric indices (height and weight) were measured according to standardized methods in the health examination. Body mass index (BMI) was calculated as body weight in kilograms (kg) divided by the square of the height in meters (m^2^).

### Blood biochemical analysis

Blood samples were obtained from each participant during the health examination. Collected specimens were centrifuged within an hour of sampling and stored at –80°C until assessment. Other biochemical analyses of blood were conducted at the laboratory of Yakumo General Hospital (Hokkaido, Japan).

### *TXNIP* DNA methylation data

Genomic DNA was extracted from peripheral blood samples using NucleoSpin Tissue kits (TaKaRa, Japan) according to the manufacturer’s instructions. The extracted DNA was bisulfite-converted with the EpiTect Fast DNA Bisulfite Kit (QIAGEN, Germany) according to the manufacturer’s protocol. For each sample, a polymerase chain reaction (PCR) was performed in a 20-μL reaction mixture containing 20 ng (in 2 μL) bisulfite-treated genomic DNA, dNTPs, TaKaRa EpiTaq HS (for bisulfite-treated DNA), MgCl_2_, the forward and reverse primers, and EpiTaq PCR buffer. The PCR-amplified DNA sequencing (chr1: bp 145,441,434–663) is shown in S1 Fig. After PCR amplification, the differential methylation at a CpG site (chr1: bp 145,441,552) within the 3’-untranslated region (3’-UTR) of *TXNIP* locus, the most frequently reported CpG site in previous studies related to *TXNIP* DNA methylation [5, 6], was validated using PyroMark Q24 Advanced (QIAGEN) amplification with a sequencing primer (5’-GGGTTAGGTAAAAATGG-3’). The *TXNIP* DNA methylation level was calculated as the percentage of methylated cytosine using the height of the T and C peaks at the methylation site.

### Statistical analysis

All statistical analyses were performed using JMP software (version 12.0; SAS Institute Inc., Cary, NC, USA). Normally distributed variables are presented as mean ± standard deviation (SD). Continuous variables were compared across smoking habits using the Analysis of Variance (ANOVA) and Tukey-Kramer HSD tests. Categorical variables were compared using a χ^2^ test. The correlations between the *TXNIP* DNA methylation levels and current smoking status, including the number of cigarettes per day, years of smoking, and the BI, were assessed using Spearman’s rank correlation analysis, because all indices of smoking status were lognormally distributed. Hypomethylation of the *TXNIP* gene was defined as frequencies of *TXNIP* gene methylation that were below the median value (77.7%). Odds ratios (ORs) and 95% confidence intervals (CIs) for hypomethylation of the *TXNIP* gene were estimated by a logistic regression analysis. We calculated the ORs for hypomethylation of the *TXNIP* gene among those with specific smoking habits using the never smokers as the reference group. Current smokers were categorized according to the number of cigarettes per day (<20 and ≥20), years of smoking (<35 and ≥35), and BI (<600 and ≥600). Current smokers were categorized based on the median values of the number of cigarettes per day and the number of years of smoking. The literature defines subjects with BI ≥ 600 as heavy smokers with associated increased risk for lung cancer and metabolic syndrome [27]; therefore this value was used as the cut-off value for categorization of current smokers by BI. We calculated the ORs for hypomethylation of the *TXNIP* gene by current number of cigarettes per day, years of smoking, and BI using the non-smokers (never and ex-smokers) as a reference. We used sex, age, BMI, hemoglobin A1c (HbA1c), high-density lipoprotein (HDL) cholesterol, alcohol consumption, and percentage of neutrophil as confounding factors. A *p-*value of less than 0.05 was considered statistically significant.

## Results

Table 1 shows the basic characteristics of study subjects according to smoking habits. Of 417 participants, 203 (48.7%) were never smokers, 149 (35.7%) were ex-smokers, and 65 (15.6%) were current smokers. Current smokers were significantly younger and had significantly lower serum levels of HDL cholesterol than did never smokers. Ex-smokers had significantly lower serum levels of HDL cholesterol compared to never smokers.

**Table 1:** Characteristics of the study subjects according to smoking habits.

		Never	Ex	Current	*p*
*n*		203	149	65	
Men, n (%)		30 (14.8)	106 (71.1)	44 (67.7)	< 0.001^b^
Age (year)^a^		64.1 ± 10.0^f^	63.1 ± 9.1^f^	58.0 ± 9.7	< 0.001^c^
Body mass index (kg/m^2^)^a^		23.3 ± 3.5	24.1 ± 3.1	23.8 ± 3.3	0.065^c^
Hemoglobin A1c (%)^a^		5.7 ± 0.5	5.8 ± 0.6	5.8 ± 0.7	0.618^c^
LDL cholesterol (mg/dL)^a^		126.4 ± 30.9	123.0 ± 27.6^d^	134.1 ± 37.8	0.056^c^
HDL cholesterol (mg/dL)^a^		62.2 ± 14.4^e^^,^^f^	57.5 ± 13.1	53.6 ± 11.8	< 0.001^c^
Percentage of neutrophil (%)^a^		54.8 ± 8.2	54.8 ± 8.2	55.0 ± 7.5	0.977^c^
Alcohol consumption, n (%)	Never	143 (70.4)	43 (28.9)	24 (36.9)	< 0.001^b^
	Ever	0 (0.0)	7 (4.7)	1 (1.5)	
	Current	60 (29.6)	99 (66.4)	40 (61.6)	

Abbreviations: LDL, low-density lipoprotein; HDL, high-density lipoprotein.

^a^ Mean ± SD.

^b^ χ^2^ test.

^c^ ANOVA.

^d^ *p* < 0.05 (vs. Current smoker; Tukey-Kramer HSD tests).

^e^ *p* < 0.01 (vs. Ex-smoker; Tukey-Kramer HSD tests).

^f^ *p* < 0.01 (vs. Current smoker; Tukey-Kramer HSD tests).

**Fig 1:**
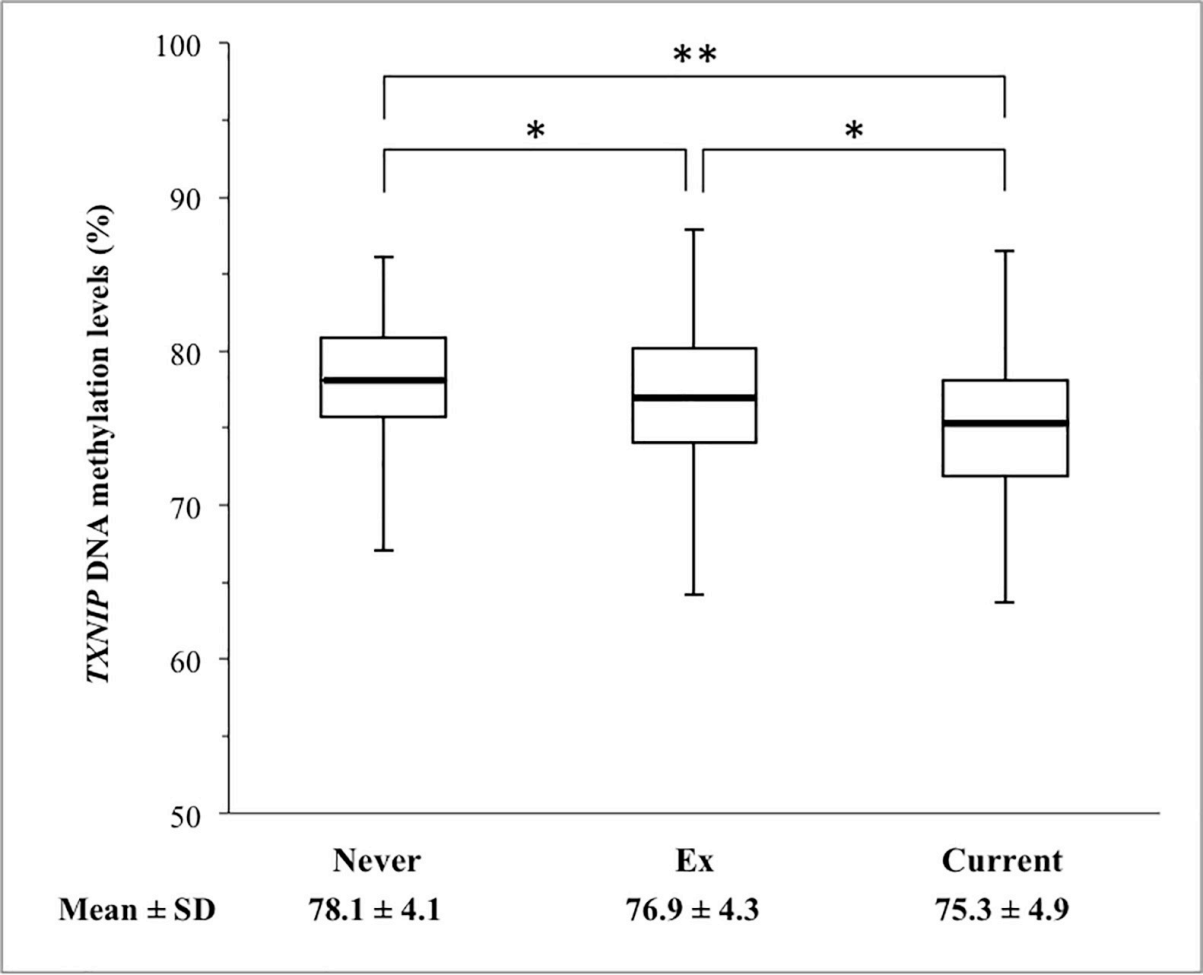
Comparison of *TXNIP* DNA methylation levels according to smoking habits. Boxplots (bold horizontal line: mean; box: interquartile range; upper whisker: maximum value; lower whisker: minimum value) of *TXNIP* DNA methylation levels in never smokers (n = 203), ex-smokers (n = 149), and current smokers (n = 65). *p < 0.05, **p < 0.01 (Tukey-Kramer HSD tests).

Fig 1 shows the mean *TXNIP* DNA methylation levels according to smoking habits. The mean *TXNIP* DNA methylation levels in current smokers (mean level ± SD: 75.3 ± 4.9%) were significantly lower than those of never smokers (mean level ± SD: 78.1 ± 4.1%, *p* <0.001) and those of ex-smokers (mean level ± SD: 76.9 ± 4.3%, *p* = 0.013). The *TXNIP* DNA methylation levels in ex-smokers also were significantly lower than those of never smokers (*p* = 0.010).

Table 2 shows Spearman’s rank coefficients for the associations between *TXNIP* DNA methylation levels and smoking status. The *TXNIP* DNA methylation levels were significantly and negatively correlated with the number of cigarettes per day (*r*_s_ = -0.187, *p* <0.001), number of smoking years (*r*_s_ = -0.187, *p* <0.001), and BI (*r*_s_ = -0.189, *p* <0.001). In men, the *TXNIP* DNA methylation levels were negatively associated with the number of cigarettes per day (*r*_s_ = -0.210, *p* = 0.005), number of smoking years (*r*_s_ = -0.221, *p* = 0.003), and BI (*r*_s_ = -0.209, *p* = 0.005); in women, these associations did not achieve statistical significance.

**Table 2:** Spearman’s rank correlation coefficients for the associations between *TXNIP* DNA methylation levels and smoking status.

		Number of cigarettes per day		Years of smoking		Brinkman Index	
	*n* (%)	*r*_s_	*p*	*r*_s_	*p*	*r*_s_	*p*
Total	417	-0.187	< 0.001	-0.187	< 0.001	-0.189	< 0.001
Men	180 (43.2)	-0.210	0.005	-0.221	0.003	-0.209	0.005
Women	237 (56.8)	-0.044	0.499	-0.044	0.545	-0.044	0.499

Abbreviations: TXNIP, thioredoxin-interacting protein; *r*_s_, Spearman’s correlation coefficient.

Table 3 shows the crude and multivariable-adjusted ORs and 95% CIs for hypomethylation of the *TXNIP* gene according to smoking habits. Significantly higher crude ORs for *TXNIP* DNA hypomethylation were observed in ex-smokers (OR, 1.88; 95% CI, 1.23–2.90) and current smokers (OR, 3.72; 95% CI, 2.06–6.94) compared to never smokers. The multivariable-adjusted ORs for *TXNIP* DNA hypomethylation were significantly higher in current smokers compared to never smokers (OR, 2.09; 95% CI, 1.05–4.24).

**Table 3:** Odds ratios (ORs) and 95% CIs for hypomethylation of the *TXNIP* gene according to smoking habits.

	*n*	Crude		Multivariable adjusted^a^	
	Hypomethylation / Total	OR	(95% CI)	OR	(95% CI)
Never	80 / 203	1.00		1.00	
Ex	82 / 149	1.88	(1.23–2.90)	1.13	(0.66–1.93)
Current	46 / 65	3.72	(2.06–6.94)	2.09	(1.05–4.24)

Abbreviations: TXNIP, thioredoxin-interacting protein; 95% CI, 95% confidence interval.

^a^Adjusted for sex, age, BMI, HbA1c, HDL cholesterol, alcohol consumption, and percentage of neutrophils.

Table 4 shows the crude and multivariable-adjusted ORs and 95% CIs for hypomethylation of the *TXNIP* gene according to current number of cigarettes per day, number of years of smoking, and BI. Significantly higher crude ORs for *TXNIP* DNA hypomethylation were observed (compared to non-smokers) in current light smokers (number of cigarettes per day <20), current heavy smokers (number of cigarettes per day ≥20), current smokers with years of smoking <35, current smokers with years of smoking ≥35, and current smokers with BI ≥ 600. The multivariable-adjusted ORs for *TXNIP* DNA hypomethylation were significantly higher (compared to those in non-smokers) in current smokers with years of smoking ≥35 (OR, 2.95; 95% CI, 1.23–7.92) and in current smokers with BI ≥600 (OR, 2.28; 95% CI, 1.01–5.55).

**Table 4:** Odds ratios (ORs) and 95% CIs for hypomethylation of the *TXNIP* gene according to smoking status.

	*n*	Crude		Multivariable adjusted^a^	
	Hypomethylation/Total	OR	(95% CI)	OR	(95% CI)
**Number of cigarettes per day**					
Non-smokers	162 / 352	1.00		1.00	
1–19	18 / 27	2.35	(1.05–5.61)	1.87	(0.81–4.58)
≥ 20	28 / 38	3.28	(1.60–7.30)	2.02	(0.91–4.77)
**Years of smoking**					
Non-smokers	162 / 352	1.00		1.00	
1–34	23 / 35	2.25	(1.10–4.80)	1.37	(0.62–3.13)
≥ 35	23 / 30	3.85	(1.69–9.92)	2.95	(1.23–7.92)
**Brinkman Index**					
Non-smokers	162 / 352	1.00		1.00	
1–599	18 / 28	2.11	(0.96–4.88)	1.65	(0.72–3.95)
≥ 600	28 / 37	3.65	(1.74–8.40)	2.28	(1.01–5.55)

Abbreviations: TXNIP, thioredoxin-interacting protein; 95% CI, 95% confidence interval.

Non-smokers included never and ex-smokers.

^a^Adjusted for sex, age, BMI, HbA1c, HDL cholesterol, alcohol consumption, and percentage of neutrophils.

## Discussion

In this study, we showed that current smokers had significant DNA hypomethylation at a CpG site (chr1: bp 145,441,552), which is located within the 3’-UTR of *TXNIP*, in leukocytes compared to those in never and ex-smokers. Moreover, we observed that the *TXNIP* DNA hypomethylation was significantly associated with longer smoking histories and higher smoking exposure (as assessed by years of smoking and BI, respectively). To our knowledge, this is the first report demonstrating that smoking is associated with the *TXNIP* DNA hypomethylation in a general population.

DNA methyltransferase 1 (DNMT1) catalyzes DNA methylation and plays an important role in the process of DNA methylation. Satta et al. [28] demonstrated down-regulation of DNMT1 expression in the frontal cortex of mice injected with nicotine. Therefore, the *TXNIP* DNA hypomethylation by smoking may be caused by down-regulation of DNMT1 expression due to nicotine exposure. Other components of cigarette smoke also have been reported to be associated with altered DNA methylation. Based on genome-wide analysis of DNA methylation in relation to smoking, Zhu et al. [23] hypothesized that exposure to naphthalene, a byproduct of cigarette smoke, alters DNA methylation. Besingi et al. reported that changes in DNA methylation are not caused by the basic chemical components of tobacco, but from the burnt products generated during the smoking process. Several of the chemical components contained in cigarette smoke are known to be key factors in DNA methylation changes [28], but the mechanistic details of the association of smoking with DNA methylation remain unclear.

Previous studies have reported association of *TXNIP* DNA methylation levels and mRNA expression levels [29]. They demonstrated that *TXNIP* DNA methylation levels were inversely correlated with TXNIP mRNA expression. Furthermore, it has been reported that the 3’-UTR region of the TXNIP mRNA contains regulatory regions that exert post-transcriptional effects on gene expression [30]. Therefore, we infer that DNA hypomethylation within the 3’-UTR of *TXNIP* may contribute to increases in mRNA levels of TXNIP. However, no previous study has been published on whether TXNIP mRNA levels are altered in smokers compared to nonsmokers. Meanwhile, a previous study reported that the p38 mitogen-activated protein kinase (MAPK) pathway up-regulated TXNIP expression through increasing TXNIP mRNA [31]. In addition, an in vivo study demonstrated that p38 was significantly increased by exposure to tobacco smoke, indicating the activation of MAPK pathway [32]. Therefore, we speculate that tobacco smoke may activate MAPK pathway and then increase mRNA levels of TXNIP in current smokers. One of the mechanisms underlying this biological pathway is likely involved in DNA hypomethylation in *TXNIP*. TXNIP down-regulates the expression and function of TXN [3]. Notably, an animal model study found that ROS induce the dissociation of TXNIP from TXN and allows TXNIP to bind to NOD-like receptor protein 3 (NLRP3), leading to NLRP3 inflammasome activation under conditions of increased oxidative stress [33]. The signaling pathway controlling the NLRP3 inflammasome is a major mediator of immune response following exposure to cigarette smoke [34]. We suggest that the *TXNIP* DNA hypomethylation associated with smoking is involved in the development of smoking-related pathologies such as cancer and cardiovascular diseases, and is mediated through the TXNIP-NLRP3 interaction.

Current smokers with longer smoking histories and higher smoking exposure had significantly lower *TXNIP* DNA methylation compared to non-smokers. Previous literature also has reported associations between DNA methylation and smoking status [35, 36]. Specifically, some CpG sites exhibit decreased methylation in current smokers, with decreasing methylation seen with increasing smoking exposure [35]. Consistent with our results, another publication reported hypomethylation of various genes in current smokers, with the DNA methylation levels showing an inverse correlation with the number of pack-years [36]. On the other hand, the present study did not detect a significant association between the degree of *TXNIP* DNA methylation and the current number of cigarettes per day. We suggest that hypomethylation of the *TXNIP* gene is more strongly affected by years of smoking and lifetime cigarette consumption than by the current amount of cigarette smoking.

Our results showed that the *TXNIP* DNA methylation levels in ex-smokers were significantly higher than those in current smokers. This finding suggests that *TXNIP* DNA hypomethylation may be counteracted by smoking cessation. Several epidemiological studies have investigated the effects of smoking cessation on DNA methylation [22, 37]. Those studies showed that smoking-associated decreases in DNA methylation at some CpG sites could be reversed by smoking cessation, while other sites remained differentially methylated. There is evidence that smoking cessation leads to improved prognoses in various pathologies such as cancer, cardiovascular disease, and respiratory disease [38]. Restoration of *TXNIP* DNA methylation by smoking cessation may contribute to the beneficial effects of quitting smoking.

Several epidemiological studies have identified associations between DNA hypomethylation in *TXNIP* and T2DM [7–10]. The authors of those studies hypothesized that changes in DNA methylation of the *TXNIP* gene might lead to failure of glucose homeostasis and a resulting increased risk of T2DM. In fact, the TXNIP protein has been reported to inhibit glucose uptake into fat and muscle and to mediate pancreatic β-cell death through apoptosis [4]. Smoking is a known risk factor for T2DM [39, 40]. The *TXNIP* DNA hypomethylation may be one of the molecular mechanisms whereby smoking contributes to an increased risk of T2DM.

This study has several limitations. First, this work cannot address the possible causal relationship between *TXNIP* DNA methylation levels and smoking habits, given that this analysis was designed as a cross-sectional study. Additional longitudinal studies are needed to elucidate the possible causality of this relationship. Second, although we found that current smokers decreased levels of *TXNIP* DNA methylation compared to those in never and ex-smokers, the difference between two groups is so small (less than 3%). For example, the previous literature also estimated the difference in DNA methylation levels at the CpG site in *TXNIP* in patients with controlled and poorly controlled T2DM compared to with individuals free of diagnosed T2DM [9]. They reported that DNA methylation levels is altered by only about 5% among controlled and poorly controlled T2DM patients compared to those no T2DM. Another previous research has also reported that poorly controlled T2DM group was hypomethylated compared with good controlled T2DM group with a mean *TXNIP* DNA methylation levels difference of approximately 3% [10]. Slight differences of *TXNIP* DNA methylation have also been reported in another paper [7]. Therefore, we consider that even small differences in *TXNIP* DNA methylation between different smoking habits may be clinical significant. Third, we measured DNA methylation in peripheral blood leukocytes. As methylation levels may be tissue specific, the associations that we found in the present study may not be generalizable to other tissues. However, a previous study reported that smoking alters DNA methylation patterns in lung tissue, a change that also was detectable in peripheral blood DNA [41]. Fourth, we need to consider the type of white blood cells (WBCs) used in our analysis, because DNA methylation may differ depending on the type of WBC. In the present study, we attempted to address this issue by adjusting for the percentage of neutrophils in our multivariable analysis. Several DNA methylation studies in peripheral blood also perform statistical analyses using percentage of neutrophil as confounding factors [42, 43]. In addition, the WBC differential count is not an estimated value, it is actually measured from each blood sample by an automated hematology analyzer LH755 (Beckman Coulter, USA). Fifth, the data on smoking history were based on patient recollections of smoking. Although those data were obtained by trained public health nurses at the health examination, criticism about the reliability of data could not be dismissed.

In conclusion, we found that leukocytes from current smokers had decreased levels of *TXNIP* DNA methylation compared to those from never and ex-smokers. Long-term smoking and high smoking exposure also were associated with DNA hypomethylation in *TXNIP*. Moreover, DNA methylation of the *TXNIP* gene may be reversed by smoking cessation. Further longitudinal studies using a larger population would clarify the possible causal nature of these associations.

## Supporting information

S1 Fig The PCR-amplified DNA sequencing and the analyzed CpG site position. A part of 3’-untranslated region (3’-UTR) of *TXNIP* locus. The PCR-amplified DNA sequencing is in bold type. Arrows show PCR primers. The number 1 represents the analyzed CpG site.
(TIFF)
