# Supplementary figures and images for: Association of smoking habits with TXNIP DNA methylation levels in leukocytes among general Japanese population
Source: PLoS One. 2020 Jul 1;15(7):e0235486. doi: 10.1371/journal.pone.0235486 (PMC7329107; doi:10.1371/journal.pone.0235486)

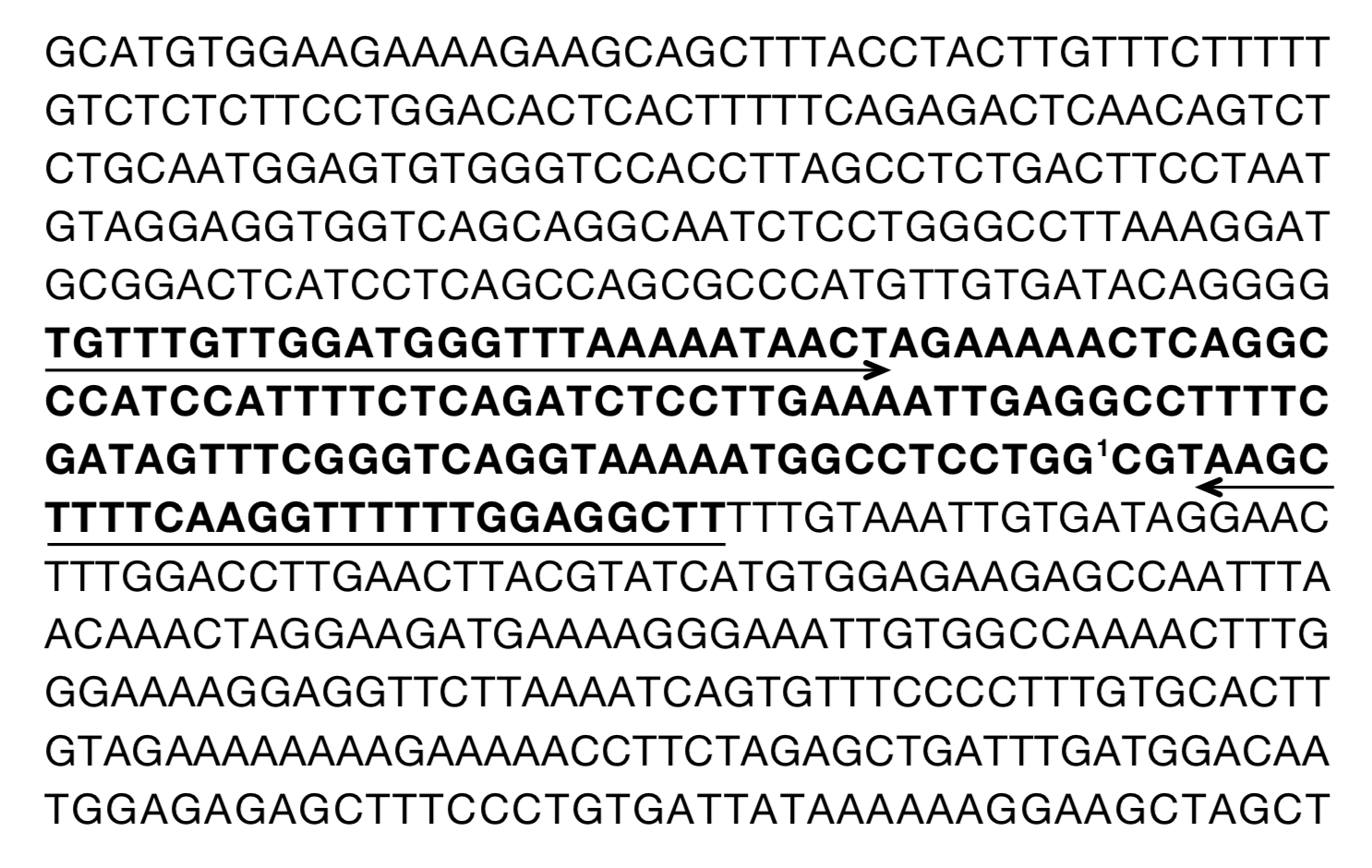

Supplement: S1 Fig — A part of 3’-untranslated region (3’-UTR) of TXNIP locus. The PCR-amplified DNA sequencing is in bold type. Arrows show PCR primers. The number 1 represents the analyzed CpG site. (TIFF) [file pone.0235486.s001.tiff]
